# Outrunning damage: Electrons vs X-rays—timescales and mechanisms

**DOI:** 10.1063/1.4984606

**Published:** 2017-06-01

**Authors:** John C. H. Spence

**Affiliations:** Department of Physics, Arizona State University, Tempe, Arizona 85282, USA

## Abstract

Toward the end of his career, Zewail developed strong interest in fast electron spectroscopy and imaging, a field to which he made important contributions toward his aim of making molecular movies free of radiation damage. We therefore compare here the atomistic mechanisms leading to destruction of protein samples in diffract-and-destroy experiments for the cases of high-energy electron beam irradiation and X-ray laser pulses. The damage processes and their time-scales are compared and relevant elastic, inelastic, and photoelectron cross sections are given. Inelastic mean-free paths for ejected electrons at very low energies in insulators are compared with the bioparticle size. The dose rate and structural damage rate for electrons are found to be much lower, allowing longer pulses, reduced beam current, and Coulomb interactions for the formation of smaller probes. High-angle electron scattering from the nucleus, which has no parallel in the X-ray case, tracks the slowly moving nuclei during the explosion, just as the gain of the XFEL (X-ray free-electron laser) has no parallel in the electron case. Despite reduced damage and much larger elastic scattering cross sections in the electron case, leading to not dissimilar elastic scattering rates (when account is taken of the greatly increased incident XFEL fluence), progress for single-particle electron diffraction is seen to depend on the effort to reduce emittance growth due to Coulomb interactions, and so allow formation of intense sub-micron beams no larger than a virus.

## I. INTRODUCTION

Zewail's remarkable career, establishing the field of femtochemistry, is described elsewhere in this volume. I first encountered his work in the late 1990s when his interests turned to pump-probe fast electron diffraction experiments, motivated, as he told me, by the strength of the electron interaction with matter. This resulted in a series of visits to his labs and much stimulating discussion with him and his excellent group working on fast electron diffraction. And it was a stimulating experience to edit an early draft of his book with Thomas on fast electron microscopy (Zewail and Thomas, 2010). After teaching quantum optics, and now more fully involved with the pump-probe X-ray laser community, my perspective on the strengths and weaknesses of these various interactions has improved a little. Perhaps, this contribution can then be thought of as continuing our discussions on a topic close to his heart, sadly one-sided in lacking his sharp responsive intelligence and continuous font of novel ideas.

Recently, we have seen the first publication of “molecular movies” from hydrated proteins with atomic resolution and femtosecond time resolution. The reaction is triggered by light (such as the cis-trans isomerization reaction (Pande *et al.*, 2016), which occurs as the first event in human vision, and photosynthesis, or, for the slower, crucial reactions of enzymatic catalysis (Kupitz *et al.*, 2017) triggered by chemical mixing. These exciting developments have opened up a new field for the study of hydrated protein dynamics, in which processes can be imaged at room temperature (without the need for cooling to reduce damage) under near-physiological conditions, and *in the correct thermal bath* in which molecular machines must operate. They are made possible by the ability of femtosecond pulses to out-run the effects of radiation damage, using pulses as brief as 10 fs or less, as discussed below. A few provisos are needed, however, to clarify the widely used jargon in this field for a wider audience. “Molecular movies” in this context are mainly obtained from time-resolved diffraction data, which, when obtained from microcrystals (in order to provide atomic resolution), require ensemble-averaging effects to be disentangled. [Time-resolved XFEL (X-ray free-electron laser) diffractive imaging from single particles at lower resolution will soon appear, which may not.] “Outrunning damage” usually refers to the elimination of secondary “impact ionization,” not the immediate “electronic damage” due to the ionization of a small fraction of the atoms in a microcrystal in the first ten femtoseconds, while “near-physiological” conditions recognizes that the volatile buffer used for crystallization is not identical to the cytoplasm in which many proteins function within a cell.

Two groups have championed the use of sub-picosecond electron beams rather than X-rays, in view of their lower cost, compact apparatus, and hence greater user access. One, of which Ahmed was a true pioneer, works with kilovolt energy beams in transmission electron microscopes fitted with a photocathode for stroboscopic imaging, with all the advantages of full-field magnetic-lens imaging and efficient energy-loss spectroscopy. Speed, however, is then reduced due to Coulomb interactions at the many high current-density beam focii needed for imaging when using magnetic lenses. Here, Zewail's discovery of the fascinating photon-induced near-field electron microscopy effect (which avoids damage), together with his use of few-electron pulses (and very high repetition rate) in order to minimize Coulomb interactions, has produced a growing body of research. A major advance also occurred with the development of the dynamic transmission electron microscope instrument at Lawrence Livermore Laboratories around 2006 [see Campbell *et al.* (2014) and Bostanjoglo (2002)], where the emphasis, by contrast, was on single-shot TEM imaging. These machines typically provide single-shot full-field real-space images with nanosecond time resolution and nanometer spatial resolution.

The second group works with MeV electron linear accelerator (LINAC) (also used to drive XFEL undulators) for electron diffraction (Weathersby *et al.*, 2015; Zhu *et al.*, 2015; and Murooka *et al.*, 2011). For both groups, the effects of Coulomb interactions (which fall off with increasing beam energy) make it difficult to achieve sub-100 fs duration pulses [but see Maxson *et al.* (2017)] and sub-micron beam diameters. In this paper, we consider differences in the time response of electron and X-ray interactions with matter which affect the prospects for out-running radiation damage using femtosecond high-energy electron beams, a subject close to the heart of Ahmed's interests.

## II. COMPARISON OF X-RAY AND ELECTRON INTERACTIONS

### A. Elastic and inelastic atomic scattering cross sections

Because of the orthogonality of initial and final atomic electron states in the Bethe scattering theory (Inokuti, 1971), inelastic electron scattering from atoms does not involve interaction with the nuclear potential, as is also the case for elastic X-ray scattering. Elastic electron scattering does, however, interact with the nucleus, suggesting that by using this nuclear scattering, the slower response of massive nucleii may allow longer times before the onset of significant nuclear motion. To separate these nuclear and electronic contributions, the differential cross section for elastic electron scattering from an atom may be written in terms of the X-ray atomic scattering factor *f*_X_(**q**) (the Fourier Transform of the atomic charge density) as (Zuo and Spence, 2017)

$$\frac{d\sigma }{d\Omega }=\frac{4\gamma^{2}}{a_{o}^{2}q^{4}}{\left[Z-f_{X}(q)\right]}^{2}.$$ (1)

Here, γ is the Lorentz factor (γ = 6.88 at 3 MeV), Z atomic number, a_o_ = 0.053 nm, and |q| = 2π Θ/λ for the small scattering angles Θ involved in electron diffraction, with λ the relativistic electron wavelength. Here, the first term describes Rutherford scattering of electrons from the slowly moving nucleus, and the second the electronic scattering which also describes X-ray scattering. The q^−4^ term amplifies electron scattering at low angles relative to X-ray scattering (which tends to Z for small angles) making it extremely sensitive to the form of the chemical bonds between atoms (Zuo *et al.*, 1999). During femtosecond pulses, the second term describes this low-angle scattering from ions, which rises strongly around **q** = 0 due to the **q**^4^ term in the denominator. High angle electron scattering from the nucleus alone, during the particle explosion, will be free of the effects of ionization beyond a scattering angle Θ_c_ ∼ λ/$\sqrt{\left\langle {\text{u}}^{2}\right\rangle }$ (defined below), falling off as q^−4^. However, the finite mass of the electron and large momentum transfers possibly mean that nuclear recoil may be important for electron-nuclear scattering, unlike the X-ray case, where momentum transfer to electrons is small (for near “vertical” transitions). The electron-nuclear scattering is the basis of the high-angle annular-detector dark field (HAADF) imaging method in scanning transmission electron microscopy (STEM), where only these electrons, due to the first term on the right-hand side of Eq. (1), are detected to form an image as the probe is scanned (Howie, 1979 and Amali and Rez, 1997).

The multiphonon diffuse scattering due to atomic vibration (in an Einstein model) which develops initially during fast intense electron irradiation is given by (Hall and Hirsch, 1965)

$$\frac{d\sigma }{d\Omega }=\frac{4\gamma^{2}{\left[Z-f_{X}(q)\right]}^{2}}{a_{o}^{2}q^{4}}[1-\text{exp}\;(-Mq^{2})].$$ (2)

Here, M = 0.5 ⟨u^2^⟩ is a temperature factor, with ⟨u^2^⟩ the mean-squared atomic vibration amplitude. This diffuse scattering is seen in individual XFEL snapshots, via the ergodic theorem, and is also present in crystals consisting of molecules with displacive disorder, as discussed below (Ayyer *et al.*, 2016).

At large scattering angles, the electronic X-ray scattering term in Eq. (1) falls to zero and the high-angle electron scattering takes on the form of Rutherford scattering from the nucleus, corresponding to small impact parameters. To determine the beam-energy dependence of the total elastic scattering cross section, we may apply a Yukawa potential to Rutherford's model, with a suitable screening radius to obtain the relativistic result (Lenz, 1954)

$$\sigma_{t}=\frac{4\pi \hbar^{2}}{m_{o}^{2}v^{2}}Z^{4/3}.$$ (3)

Since the electron velocity rapidly approaches a limit, neither this cross section, nor that for inelastic scattering from atomic electrons (with similar beam energy dependence) vary much with beam energy above 1 MeV. Increased beam energy cannot therefore be used to discriminate against unwanted background due to inelastic electron scattering and the resulting radiation damage; however, it does allow the use of thicker samples, since it delays the onset of multiple elastic scattering perturbations.

### B. Inelastic scattering and stopping power

**FIG. 1.**
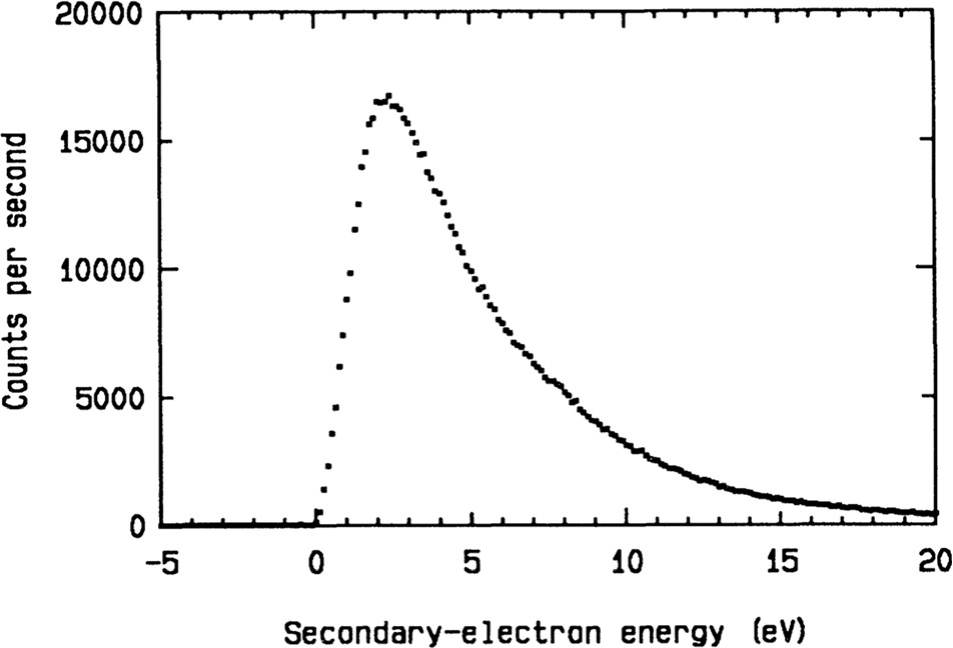
Energy spectrum of secondary electrons ejected from a thin amorphous carbon film. Reprinted with permission from Pijper, F. and Kruit, P., Phys Rev. B **44**, 9192 (1991). Copyright 1991 by American Physical Society.

**FIG. 2.**
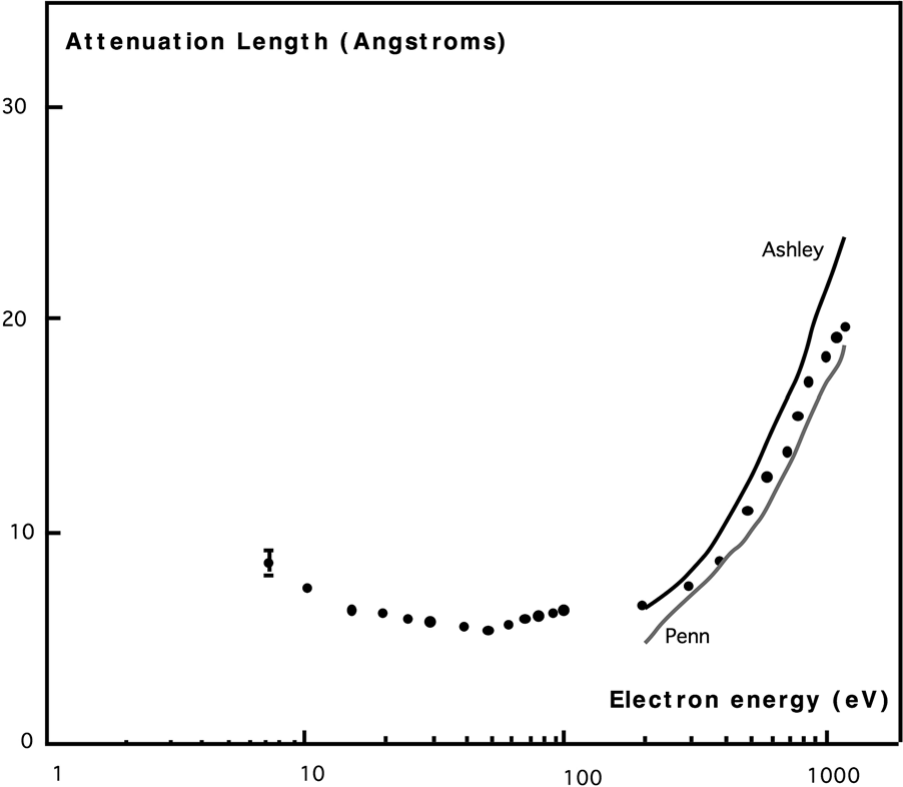
Attenuation length for electrons traversing a 4 nm thick carbon film as a function of beam energy, measured using an energy-loss spectrometer. Continuous lines are theoretical predictions (Tanuma *et al.*, 2005). Republished with permission from Martin *et al.*, J. Electron Spectrosc. Relat. Phenom. **42**, 171 (1987). Copyright 1987 by Elsevier (Martin *et al.*, 1987).

The electron charge and mass produce fundamentally distinct interactions from the X-ray beam case. As with X-rays, however, strong electric fields and resulting forces from the tube of charge surrounding the electron beam are known to displace atoms in insulators, unlike metals where rapid screening occurs, minimizing damage (Jiang, 2016). Electron-beam excitations may be classified as ballistic “knock-on” events, or inner-shell, single-electron or collective excitations (such as plasmons and phonons, causing heating). Other processes include hole-drilling, sputtering, and mass-loss. “Single-electron” excitations refer to valence electrons directly ejected by the electron beam. Beam electrons are not annihilated in these interactions, unlike X-rays, but continue to the detector to produce background. Ejected valence electrons are described as “secondary electrons,” and this process has a much higher cross section (comparable to the cross section for elastic scattering) than ejection of inner-shell electrons or collective excitations for the light atoms in proteins (Reimer and Kohl, 2008). Inner-shell excitations, although less frequent than valence excitations for light elements, deposit more energy in the sample, increasing their contribution to stopping power. The relative contributions of these processes can be judged directly from the features in energy-loss spectra from proteins (Sun *et al.*, 1993 and Egerton, 2011). We will therefore neglect all processes except valence electron excitation, which has been extensively studied by energy-loss spectroscopy in TEM, interpreted using the dielectric response theory (Howie, 2003). Direct detection of these low energy secondary electrons is also possible using an electron spectrometer. Figure 1 shows measurements of the energy distribution of secondary electrons ejected from a thin carbon film traversed by an 80 kV electron beam (Pijper and Kruit, 1991). A weak peak is also seen at 260 eV (not shown) corresponding to the carbon KVV Auger process. The peak of this distribution occurs at about 3 eV (120 kT), corresponding to a secondary electron velocity within the sample of 1 nm/fs., requiring 100 fs to traverse a 100 nm virus. Figure 2 shows measurements of the inelastic mean free path (IMFP) of electrons as a function of electron beam energy for a thin carbon film, measured using hemispherical energy-loss spectrometers, one to monochromate the incident beam and a second to isolate the transmitted elastic intensity after passing through a carbon film 4 nm thick. This suggests an inelastic mean free path of about 1 nm at 3 eV. Emerson *et al.* (1973) confirm that the largest contribution to the stopping power of 100 kV electrons in light elements arises from electrons just below the valence shell.

The measurement and theoretical treatment of IMFP for electrons at these very low energies involves many complexities and becomes highly dependent on the electronic structure of the sample. Not all inelastic scattering events result in damage in the sense of an irreversible displacement of the nucleus, which, unlike X-rays, scatters an electron beam. [At higher energies the well-established “universal curve” used in surface science can be used (Tanuma *et al.*, 2005).] The IMFP shows a minimum near the plasmon energy, increasing below it, as fewer excited electronic states become available to an electron with decreasing energy, as shown in Fig. 2. Since 3 eV corresponds to the ultra-violet range of the spectrum, we might expect roughly similar damage effects to ultraviolet light. Electrons for which the sum of kinetic energy E_K_ (measured from the vacuum level) and the electron affinity ϕ_Α_ is less than the band gap cannot lose any energy to electronic processes, and so can be expected to have a long IMFP. [This is because the maximum amount of energy (E_K_ + Φ_A_) which the electron can lose is insufficient to promote an electron across the gap.] This situation may obtain for some phases of ice, which has a large bandgap and small affinity.

The radiology community has recently devoted considerable effort to the measurement of the electron IMFP at few-eV energies by the LEET (low energy electron transmission) method, in which an organic layer is added to a metal substrate in which electrons are photoexcited by X-ray photo-electron spectroscopy and ultraviolet photoelectron spectroscopy. The electron transmission through the film is measured as a function of film thickness using an electron spectrometer. (The films are evidently not hydrated.) The aim is to understand the damage mechanisms of low energy electrons, since it has been discovered (Boudaiffa *et al.*, 2000) that even below the ionization threshold for DNA at 7 eV, electrons can be captured into a resonant state, leading to dissociation. Caron *et al.* (2008) give calculated cross sections for electron scattering from DNA in the range 0–10 eV and references to earlier work on proteins by the Sanche group, which has published LEET data for a wide range of organics. For long-chain alkanes, IMFP and energy-loss spectra have been measured in the 0.1–4500 eV range using LEET by Cartier *et al.* (1987), where the ratio of elastic to inelastic scattering cross sections can be found with a diagram of energy levels.

The most direct evidence of structural damage at low electron energies comes from Low Energy Electron Diffraction (LEED), where the fading of outer Bragg spots can be timed for a given dose. Since insulating solids retain charge, making them unsuitable as LEED samples, few results have been published for organic films; however, work on thin coronene and ethane films on a conductive substrate at 50 eV indicates that the results are highly materials specific (Zimmerman and Karl, 1992). Aromatic pi-bonded systems are well known to be more damage resistant than aliphatics, with large G-factors. The low doses used in these LEED studies are comparable with those used in Cryo-em (about 1 electron per square Angstrom). The collection of LEED data at energies below 10 eV creates serious experimental difficulties. Additional evidence comes from energy-loss studies in the reflection geometry (HREELS), where studies of benzene give an IMFP of 9 nm at 4 eV (Goulet and Pou, 1986). A survey of this literature gives IMFP values between 1 and 100 nm around 3 eV for hydrocarbons, depending on the sample.

The ballistic “knock-on” event, in which a head-on collision between beam electron and nucleus results in irreversible nuclear displacement, has been well studied in the large literature on radiation damage to nuclear reactor vessels, where it causes swelling due to Frenkel pair formation. The number of displaced atoms per second per atom is C_d_ = j σ_d_/|e| for current density j. The cross section σ_d_ ∼ 133b for carbon at 0.5 MeV (Dietrich *et al.*, 1978), which is small compared to the elastic and inelastic cross sections given above.

Electron-phonon scattering produces a heating effect, which, in XFEL experiments, does not have time to dissipate. Full-field energy-filtering devices, such as an Omega filter, which act as high-quality lenses, have been built for TEMs up to about 1 MeV; however, their energy resolution of about 1 eV at high beam energies could not at present discriminate against the inelastic phonon scattering background. At kilovolt beam energies, serial-detection filters have achieved millivolt resolution, providing phonon energy-loss spectroscopy.

### C. Diffract-and-destroy probabilities

From the beginning, radiation damage has limited resolution in practically all forms of microscopy and diffractive imaging. Historically, the assumption has always been made that the radiation dose needed to image one isolated molecule using any form of long-lived radiation (except neutrons, for which bright sources did not exist) would destroy it (Breedlove and Trammel, 1970). In an influential paper, Henderson (1995) compared X-ray and electron beams for imaging the atomic structure of organic molecules by using cross sections σ to compare the amount of useful image-forming elastic (coherent) scattering to the damage done by inelastic scattering in the two cases. His metric R = (σ_in_/σ_el_) ΔE, giving the amount of energy deposited in the sample by inelastic processes (such as the photoelectric effect) per unit elastic scattering, was found to strongly favor electrons (R_e_ = 60, σ_in_ ∼ 3 × 10^6^ barn, σ_el_ ∼ 1 × 10^6^ barn, ΔE = 20 eV) over X-rays (R_X_ = 8 × 10^4^, σ_in_ ∼ 20b, σ_el_ ∼ 2b, ΔE = 8 kV) . The cross sections are given for 0.1 MeV electrons (these vary relativistically as the inverse square of beam-electron velocity) and for 8 kV photons. R_e_ is approximately independent of beam energy, while R_X_ varies strongly with beam energy as processes other than the photoelectric effect (Compton and Auger) become important. This result (R_e_ ≪ R_X_) reflects the very large photoelectric cross section for X-rays relative to that for coherent X-ray scattering, and the large average amount of energy ΔE dumped as a result. For XFEL diffraction from submicron protein nanocrystals (and single particles, such as a virus), however, most photoelectrons escape from the sample rather than depositing their energy in it, whereas the much shorter inelastic mean free path for the few-eV electrons excited by an electron beam is less likely to escape. Thus, the fraction of energy deposited in the sample by ejected electrons can provide an important point of comparison, in addition to the Bragg boost discussed below, both of which depend on the microcrystal size. The value of R_x_ should be compared to an elastic-to-inelastic scattering ratio for electrons nearer unity, a much larger elastic scattering cross section, and more modest amount of energy deposited per inelastic event. In protein crystallography, over 98% of 12 kV photons pass through a typical crystal without interaction. Of the remaining 2%, 84% are annihilated in production of photoelectrons, 8% are scattered by the Compton process, and 8% are Bragg scattered. By comparison, in rough qualitative terms, for 0.5 MeV electrons, a low atomic number sample thickness of a micron may see the direct beam reduced to negligible intensity by elastic and inelastic scattering processes of comparable strength in the sample. Cryo-em samples for “single-particle” full-field imaging typically consist of large macromolecules, perhaps 4–20 nm in size, embedded within a slab of vitreous ice about 60 nm thick, illuminated by a collimated coherent beam of 0.4 MeV electrons.

Three factors, however, favor X-rays: (i) The source properties must be considered, and, despite the high brightness and coherence of field-emission electron sources, the new free-electron X-ray lasers (EXFEL) have gain (perhaps five or six orders of magnitude), not present in electron accelerators. (ii) X-rays which have excited photoelectrons are annihilated, unlike fast electrons, which can lose any amount of energy in the sample, after which they continue to the detector to create a large background, not seen in X-ray work. (This background can sometimes be reduced using an energy filter, as discussed above.) The X-ray diffraction patterns therefore generally show much less background than unfiltered electron diffraction patterns. (iii) The onset of multiple elastic scattering, requiring the use of samples tens of nanometers thick in TEM (thinner than a cell), is not limiting in hard X-ray studies.

Favoring TEM are the existence of high-resolution electron lenses for direct imaging (avoiding the phase problem) and the evident recent dramatic successes of the Cryo-em method with new detectors [see Spence (2013) for a review]. For pump-probe experiments, the optical pump absorption length is a closer match to electron mean-free paths than in the X-ray case; however, the use of submicron crystals in the XFEL also addresses this issue. Ultimately these differences are traceable to the different particle interactions, and the electron mass and charge. Strong interaction increases signal, inelastic scattering (and hence damage) and detection efficiency; weak scattering permits a simple single-scattering interpretation, but reduces signal and detection efficiency. Clearly sample and detector materials must differ widely.

The prospects for out-running damage in electron diffraction may be based on the published performance of existing electron accelerators, if we assume that the pulse duration needed to do so is about the same as that used with the XFEL, rather than the much longer time needed to deliver the critical dose (Spence *et al.*, 2015 and Egerton 2015). A recent 3 MeV electron diffraction camera (Murooka *et al.*, 2011) can focus 1.04 × 10^6^ electrons in a 100 fs pulse into a 500 *μ*m diameter spot with 5 × 10^−5^ radian beam divergence (providing adequate spatial coherence). This is similar performance to the SLAC ultrafast electron diffraction machine (Weathersby *et al.*, 2015). An idealized spherical viral capsid of 90 nm diameter and average protein composition then scatters less than one electron per shot, if we take the average electron cross section per effective carbon atom to be σ = 0.003 A^2^ at 100 kV (Rez, 2003), scaled by the appropriate Lorentz factor λ^2^γ^2^ ∼ 1/v^2^ for other beam energies. However, if Coulomb interactions in the electron beam could be mitigated at this energy, allowing formation of a beam diameter equal to that of the virus, with the same number of incident electrons, the number of electrons scattered in a direct hit rises to about 50 000, which may be sufficient to allow orientation-determination, merging of successive shots, phasing of data and reconstruction into a three-dimensional image (density map), if background is not limiting. The use of much higher beam energies, where electric and magnetic forces within the beam tend to cancel, is a field of active research. Simulations (Graves, 2016) predict that about 20 000 electrons would actually remain in the focus of a 7 MeV beam at 100 nm diameter, resulting in about 600 electrons elastically scattered by the virus. By comparison, in the first “single-particle” experiments at LCLS (Linac coherent light source) (Seibert *et al.*, 2011) using a coherent 1.8 kV X-ray beam (0.69 nm wavelength) focused to 10 microns, and containing about 1 × 10^12^ photons, the number of X-ray photons scattered per 70 fs pulse from a 450 nm diameter Mimivirus was about 1.7 × 10^6^. The greater number of photons scattered than electrons is primarily due to the gain of the X-ray laser, despite the much larger electron scattering cross section. In both methods, the most challenging experimental difficulty is achieving a high percentage of direct hits on a *hydrated* sample with a beam diameter about equal to the particle diameter (Ekeberg *et al.*, 2015).

## III. FORMATION OF HIGH INTENSITY FOCUSED PULSES

Unlike X-rays, the electron charge results in strong Coulomb repulsion effects in an electron beam which introduces both unwanted additional beam divergence (destroying spatial coherence) and unwanted additional energy spread (Boersch effect, destroying temporal coherence) in the beam. While these effects are negligible at the current densities used in conventional TEM (e.g., Cryo-em) with its continuous (CW) coherent electron beam, they become limiting when attempting to pack large numbers of electrons into sub-ps electron pulses focussed to micron or smaller dimensions in a linear accelerator (LINAC). The transverse emittance (loosely the product of source size and emission angle) and time resolution of X-rays, and the electron beams which generate them, are linked through the resonance condition required for X-ray “lasing” or gain, in the Self-Amplified Spontaneous Emission (SASE) mode (Pellegrini, 2012 and Clark, 2004). (This mode does not depend on stimulated emission or use a cavity as for a conventional optical laser, but does amplify noise fluctuations, and is considered a laser because it provides gain.) Meeting this lasing condition requires careful control of electron beam emittance, which tends to develop unwanted growth due to the Coulomb interactions which degrade coherence.

However, the relativistically corrected Lorentz force, in the laboratory frame, acting on one electron at **r** in a moving tube of charge density ρ is

$$F=\frac{e\rho }{2\varepsilon_{o}}(1-\beta^{2})r.$$ (5)

This tends to zero as the speed of the electron v = βc tends to the speed of light c, as the magnetic and electric forces described by the two terms on the right tend to cancel in the relativistic limit. In the rest frame of the electron, where magnetic fields are absent, the electron spends less time in the length-contracted focus at higher energy. In the lab frame, charges experience less (dilated) time as they pass through focus at higher energy. This provides the incentive for the use of higher energy beams in order to form smaller focused probes. Research into methods for emittance control is an active field of research (Batygin, 2017).

The issues involved in formation of nanometer-diameter coherent hard X-ray beams, using various forms of X-ray optics, are extensively covered in the literature (Mimura *et al.*, 2010).

## IV. ELECTRON AND X-RAY INDUCED DAMAGE PROCESSES AND THEIR TIME-SCALES COMPARED

The effects of the duration of the incident pulse and the time-evolution of the damage processes were first discussed by Breedlove and Trammel (1970), Solem (1986), Reiss (1997), Ditmire (1998), and, in detailed simulations by Neutze *et al.* (2000). As synchrotron sources and detectors improved, the possibility of out-running damage for very slow processes (such as bubble formation) became evident in protein crystallography long ago. In Cryo-em, the new fast detectors make it possible to outrun early sample movement (Glaeser *et al.*, 2011). But the invention of the hard X-ray laser (Pellegrini, 2012) has finally broken the nexus between sample size, resolution, and radiation dose (Howells *et al.*, 2009), using femtosecond X-ray pulses to generate enough elastic scattering for image formation before the onset of the photoelectron cascade which subsequently destroys the sample [see Chapman (2006) and Spence (2017) for reviews]. The gain of the SASE-mode XFEL makes this possible, with the prospect of packing a sufficient number of photons into each fs pulse to provide enough elastic scattering prior to the onset of damage to image individual molecules (Seibert *et al.*, 2011).

For proteins in the form of submicron crystals, the effective pulse duration which matters is the time until the Bragg reflections fade, which may be less than the actual pulse duration (Barty *et al.*, 2012), and one benefits from the squaring effect of the Bragg boost, in which coherent amplification gives intensity at the Bragg peak proportional to the square of the number of molecules in the crystal. This boost has a very large effect—XFEL diffraction patterns from proteins as small as 17 molecules on a side have been obtained (Chapman *et al.*, 2011). With only ten molecules on a side, the intensity at the Bragg peak (not the intensity integrated across the angular range of the peak) is therefore a million times greater than that from one molecule. This explains why atomic-resolution imaging has readily been obtained at XFELs using microcrystals, but has so far proven elusive for single-particles (with one particle, such as a virus, per shot). Bragg scattering is also special in the sense that one “knows where it is,” and can use this information to discriminate against background. For proteins containing heavy atoms with many electrons, an intense shower of photoelectrons is expected locally, so that shorter pulses (e.g., 10 fs) may be needed for high resolution. Nevertheless, using micron-sized protein crystals, atomic resolution is now routinely obtained using 40 fs pulses, whereas for single particles (e.g., one virus per shot), scattering to 0.5 nm has been recorded in rare direct hits, but resolution decreases to about 10 nm after three-dimensional reconstruction for technical reasons (Ekeberg *et al.*, 2015).

A rare opportunity to compare damage processes and their time evolution from microcrystals with those in single particles has been provided by the recent observation of XFEL diffraction from micro-crystals containing molecules with predominantly translational disorder (Ayyer *et al.*, 2016). These XFEL snapshots generate a “single-particle” pattern of diffuse scattering (except around the origin) in addition to Bragg scattering, which is an *incoherent* sum of the scattering from all the molecules in the crystal, according to Eq. (2). Diffraction patterns have been obtained for a range of pulse durations, so that the fading of both Bragg and diffuse scattering at high angles could in principle be compared for different pulse lengths. As we see from Eq. (2), the diffuse scattering at high angles is not attenuated by a temperature factor, unlike the Bragg scattering.

In protein crystallography, a “critical dose” of 30 MGy (1 Gy = 1 J/kg) is defined as the maximum tolerable for cryo-cooled samples. A dose of 1 MGy heats water from RT to a final temperature of 208 K, while the typical 1 GGy dose from an XFEL pulse eventually generates a plasma at 2 × 10^5^ K. However, fine detail is destroyed first, and late-arriving photons see a crystal with loss of short-range order, so that this dose is a function of resolution, as seen in spot-fading experiments, in which the time taken for Bragg spots to fade is measured (Howells *et al.*, 2009). At the critical dose, a cubic micron of protein crystal scatters about 1 × 10^6^ hard X-ray photons into Bragg beams. Yet only 0.06% of the atoms in an all-carbon sample would be ionized at this dose at 6 kV. A typical XFEL dose may be 1 GGy, reflecting the much higher dose possible (and hence greater signal) when using pulses which terminate before the onset of significant damage.

**FIG. 3.**
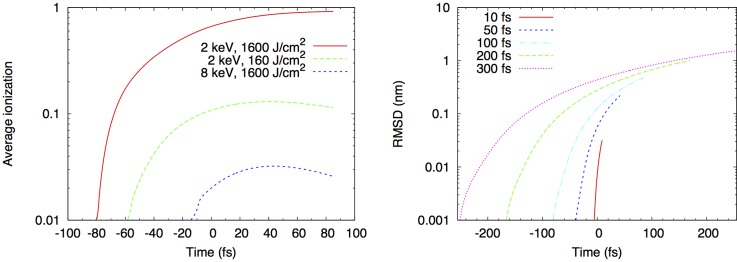
(a) Average ionization events per atom in generic protein during XFEL pulse of 100 fs. (b) Atomic displacements calculated during 2 kV pulse with 1 × 10^12^ photons into 10 *μ*m^2^ area. Gaussian pulse profile centered at t = 0. Reprinted with permission from Caleman *et al.*, J. Mod. Opt. **58**, 1486 (2011). Copyright 2011 by Taylor & Francis.

For the XFEL, the predominant damage process results from K-shell ionization of the carbon, oxygen, nitrogen, and sulfur atoms which make up proteins (Chapman *et al.*, 2014), and ejection of photoelectrons, with average energy about equal to the beam energy, and velocity about 40 nm/fs. This occurs at a rate σ_pe_ I per atom for incident photon flux I. The subsequent electronic relaxation generates, in most cases, a second Auger electron (average energy 250 eV, velocity about 7 nm/fs) from each ionization event. The carbon K hole lifetime, which controls the Auger line-widths and Auger electron emission rate per atom, is 11 fs. These electrons generate further ionization as they leave the particle, or may thermalize before doing so. For a 6 kV beam energy, the photoelectron has a mean-free path of 15 nm and thermalizes in 10 fs, producing about 240 additional ionizations, 19 of which are inner-shell. If they leave the particle, photoelectrons may return to the increasingly positively charged particle at later times. The photoelectron cascade has been analyzed in detail using both plasma and molecular dynamics algorithms, in which atoms change to ions with a given ionization probability and lifetime as time progresses, and the photoelectron cascade is tracked in Monte-Carlo simulation. For a 40 fs pulse at 1.8 keV, 10% of carbon atoms are ionized if the incident beam has irradiance 10^17^ W/cm^−2^, corresponding to about 1 × 10^12^ photons/pulse in a 7 *μ*m spot. Figure 3 shows that the average number of ionizations per atom at the end of a 100 fs LCLS pulse (1600 J cm^−2^) is about 0.9 (using 2 kV X-rays incident on protein). The RMS displacement of an atom is less than 0.1 nm at the end of a 50 fs pulse, as simulated using the modified Gromax molecular dynamics code (Caleman *et al.*, 2011). The ultimate fate of the particle depends on whether the ejected electrons thermalize (for particles larger than the inelastic mean free path of the electrons) or escape from the particle, where accumulating positive charge in the particle results in a Coulomb explosion. Photoelectrons are thus more likely to escape than Auger electrons, which have a higher cross-section for ionization, being inversely proportional to the square of velocity in the Bethe theory. This favors use of smaller samples for less damage. The importance of this sample-size effect, relative to the benefit of out-running damage using brief pulses, has not been fully investigated.

The X-ray and electron methods may also be compared by estimating the time until a certain critical amount of energy has been deposited in the sample. The dose rate (energy deposited per unit mass per unit time, in Gy/s) is D = I_o_ ΔE *ρ* σ_in_ T/M, where I_o_ is incident particle (not energy) flux, ΔE the energy deposited per interaction with cross section σ_in_, particle density *ρ*, thickness T, and particle mass M. For our 3 MeV accelerator example above (with same beam and virus size) compared to 8 kV X-rays, find that the electron dose rate (and hence the rate of structural damage) is 10^−4^ times that for X-rays.

## V. CONCLUSIONS

This discussion has assumed particle-counting detectors, which have only very recently become a possibility for XFEL work—for the more common integrating detector the detected signal depends on the product of particle number and energy deposited in the detector, with different efficiencies for high-energy electrons and photons. Our analysis is also not accurate for the analysis of coherent phase-contrast imaging, where contrast depends on scattering amplitudes, not cross-sections, and increases steadily at least up to 5 MeV (see Spence, 2013).

In summary, for equal incident particle fluence, comparing 3 MeV electrons with 8 kV X-rays, one has about 150 000 more electrons scattered elastically from carbon atoms than photons. The high-angle Rutherford electron scattering tracks the slower-moving nuclear positions throughout the explosion. The electron beam generates about 45 000 more low energy (3–5 eV) secondary electrons than high energy (8 kV) photoelectrons generated by the X-ray beam, while, in the absence of energy-filtering, the electron beam generates a larger background than the X-rays, due mainly to beam electrons which have lost energy exciting secondary (valence) electrons. For a SASE-mode XFEL supplying 1 × 10^12^ incident hard X-rays per pulse (compared with about 1 × 10^6^ electrons from a 3 MeV electron gun), the gain of the XFEL laser then compensates for the reduced amount of elastic X-ray scattering relative to that from an electron beam. Yet the dose rate is far lower for electrons, allowing longer pulses prior to damage, which thereby reduces beam current and Coulomb interactions. If the time-scale for damage is set by the critical dose, then a much longer pulse at lower beam current could be used in the example given above (Murooka *et al.*, 2011) to deliver the same dose, thereby reducing Coulomb interactions and allowing formation of a smaller beam-diameter at focus, however a penalty in time resolution results. Accurate estimates of these time-dependent and many other competing effects require molecular dynamics simulations, which are in progress. Only these can take full account fate of ionized atoms—in some cases no irreversible nuclear motion takes place after ionization, so that, if the molecular replacement method is used to phase the data, the effect of this electronic damage is not important unless very high resolution is required. (In the molecular replacement method, a model protein structure is fitted to the diffraction data to solve the phase problem). A more complete comparison should include, for the two cases, the effects of sample size relative to the IMFP, the momentum transfer and Coulomb repulsion tending to displace ionized atoms in the electron beam case, the reduced amount of energy deposited in the sample by electrons, and the differing extent of charging of the entire sample with time.

Success with electron beams is seen to lie mainly with the accelerator physics community, in the battle against the emittance growth which prevents the formation of intense submicron electron beams of comparable size to a biological target, such as a virus (perhaps 0.1 *μ*m in diameter). Such a small beam (with zero impact parameter for each shot!) will be required to provide sufficient elastic scattering for 3D image-formation.
